# Changes in molecular markers of *Plasmodium falciparum* drug resistance between 2017 and 2024 in Southern Benin

**DOI:** 10.1016/j.ijpddr.2026.100670

**Published:** 2026-09-01

**Authors:** Francis Emmanuel Towanou Bohissou, Diolinda Nahum, Juliana Inoue, Paul Sondo, Lazare Hounsou, Gethaime Sondjo, Nicaise Denis Codjo Djègbè, Robinson Woli, Salomon Dossou, Charlotte Kpohonnou, Halidou Tinto, Jana Held

**Affiliations:** a Institute of Tropical Medicine, University Hospital Tübingen, Tübingen, 72074, Germany; b Institut de Recherche en Sciences de la Santé (IRSS)/Clinical Research Unit of Nanoro (CRUN), Nanoro, Burkina Faso; c Centre de Recherche Entomologique de Cotonou (CREC), Cotonou, Republic of Benin; d German Center for Infection Research (DZIF), Partner Site Tübingen, Tübingen, Germany; e Centre de Recherches Médicales de Lambaréné, (CERMEL), Lambaréné, Gabon; f Institut de Recherche en Sciences de la Santé (IRSS)/Direction Régionale de l’Ouest, Bobo-Dioulasso, Burkina Faso

**Keywords:** *Plasmodium falciparum*, Antimalarial drugs, Resistance, Molecular markers, Benin

## Abstract

**Background:**

Benin remains highly burdened by malaria, and data on antimalarial resistance surveillance are limited. This study assesses the evolution of molecular markers associated with antimalarial resistance in southern Benin between 2017 and 2024.

**Methods:**

We analysed 358 dried blood spot samples collected from children in southern Benin (Kétonou, Kpomè and Klouékanmè) in 2017 and 2024. Molecular markers in the *PfKelch13*, *Pfmdr1*, *Pfcrt*, *Pfdhfr*, and *Pfdhps* genes associated with antimalarial drug resistance were investigated. *Pfcrt* haplotypes were characterised using quantitative PCR, while the remaining markers were analysed by nested PCR followed by Sanger sequencing.

**Results:**

None of the detected non-synonymous *PfKelch13* mutations (7/281) was a WHO-validated, candidate or potential marker of artemisinin partial resistance. The *Pfcrt* CV**IET** haplotype declined significantly from 88.1% (59/67) in 2017 to 35.4% (101/285) in 2024 (p < 0.001). The *Pfmdr1* N86**Y** decreased from 11.6% (8/69) to 6.0% (17/285), while the N**F**D haplotype increased slightly from 56.9% (37/65) to 61.9% (169/273). No mutations were observed at *Pfdhfr* I164**L** or *Pfdhps* K540**E**. The *Pfdhfr* triple-mutant **IRN**I haplotype remained nearly fixed over time at 97.0% (64/66) in 2017 vs 96.4% (268/278) in 2024. A similar trend was observed for *Pfdhps* A437**G** at 98% in 2017 and 2024. The *Pfdhps* I431**V** rose from 8.3% (5/60) in 2017 to 10.5% (29/275) in 2024, whereas *Pfdhps* A581**G** remained stable at 8%. The combined *Pfdhfr/Pfdhps***IRN**_**G** haplotype remained highly prevalent (96.5% vs 94.6%). Aside from the significant decline in CV**IET**, none of the other changes was statistically significant.

**Conclusion:**

Our findings show no mutations compromising ACT efficacy, nor any immediate threat to the continued use of SP. In addition, the observed re-emergence of chloroquine-sensitive genotypes suggests a shift in parasite population dynamics over time. Nevertheless, continuous molecular surveillance remains essential for detecting emerging resistance and informing timely policy decisions.

## Highlights

• No mutations linked to artemisinin partial resistance were identified.
• *Pfcrt* CV**IET** haplotype (chloroquine resistance) declined significantly from 2017 to 2024.
• *Pfmdr1* N**F**D haplotype, selected by artemether-lumefantrine, increased, estimated at 61.9% in 2024.
• No *Pfdhps* K540**E** mutations, associated with full sulfadoxine–pyrimethamine resistance, were detected.
• New *Pfdhps* I431V mutation reached 10.5% in 2024.

## Graphical abstract

**Figure:**
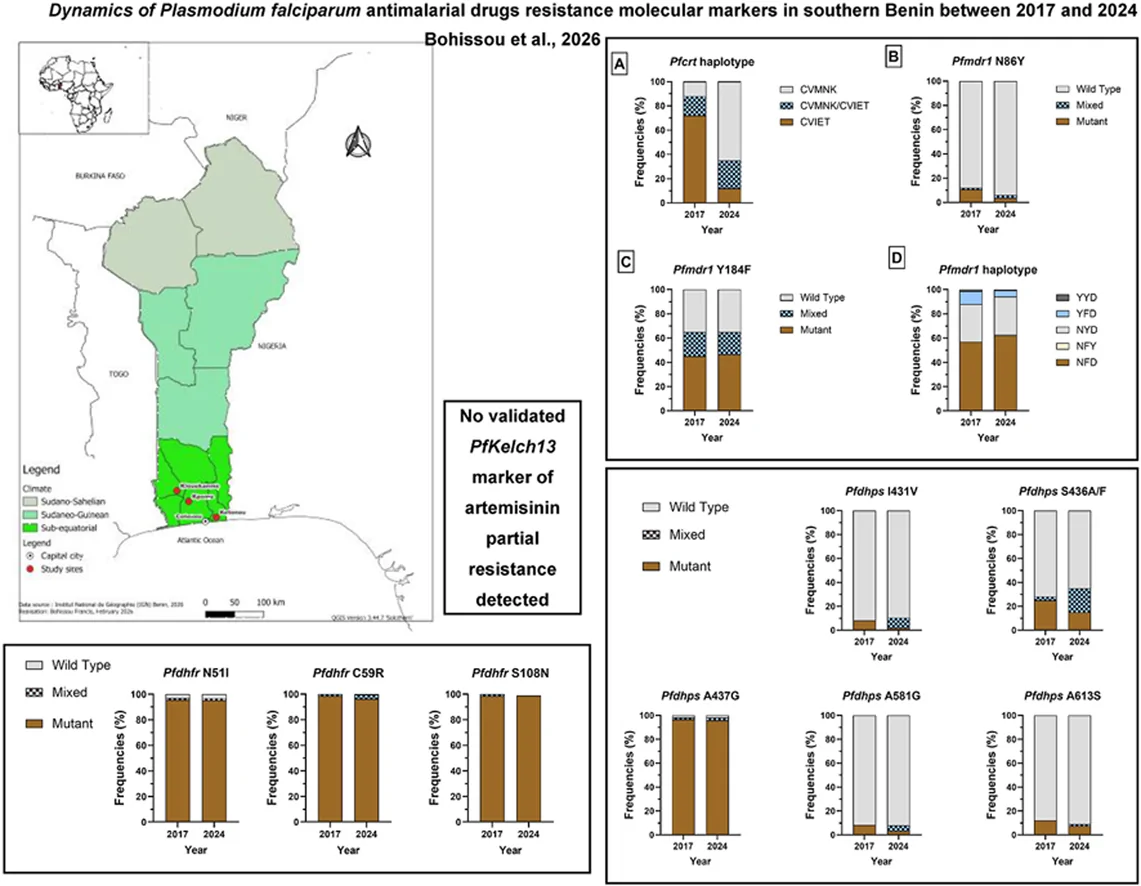

## 1 Introduction

Despite the implementation of multiple malaria control interventions, malaria remains the leading cause of hospitalisation and mortality in Benin, with an incidence estimated at 354.3 cases per 1000 population in 2024 (World Malaria Report, 2025) . During the same year, malaria accounted for 29% of consultations and hospital admissions, and 11.9 deaths per 1000 hospitalised patients (Chabi et al., 2025). This report highlights its substantial contribution to morbidity and in-hospital mortality nationwide.

To address this burden, Benin has implemented several preventive interventions, including nationwide intermittent preventive treatment in pregnancy with sulfadoxine–pyrimethamine (IPTp-SP) since 2004 (World Malaria Report, 2024), seasonal malaria chemoprevention (SMC) with sulfadoxine–pyrimethamine (SP) plus amodiaquine, for children aged 6 months to 5 years in northern Benin since 2019 and perennial malaria chemoprevention (PMC) with SP for children up to 24 months in three southern districts since 2024 (World Malaria Report, 2025). In 2024, 54.3% of pregnant women attending antenatal care (ANC) received at least three doses of SP (Chabi et al., 2025), while SMC reached 643,036 children of approximately 850,000 targeted children (Chabi et al., 2025; World Malaria Report, 2025). Malaria vaccination with RTS,S/AS01 and R21/Matrix-M was also adopted nationally in 2024, with 95,100 children aged 6 to 24 months receiving at least one dose (Chabi et al., 2025).

Since 2013, Benin has also implemented community case management of malaria, including diagnosis using rapid diagnostic tests (RDTs) and treatment with artemether–lumefantrine (AL) for children under five years of age (COSMIC Consortium, 2019). Currently, AL and artesunate–amodiaquine (ASAQ) are used as first- and second-line treatments for uncomplicated malaria (World Malaria Report, 2025). In 2025, the National Malaria Control Programme (NMCP) incorporated an additional artemisinin-based combination therapy (ACT), artesunate pyronaridine, for the treatment of uncomplicated malaria, in line with World Health Organisation (WHO) recommendations (Malaria guidelines, 2025).

The efficacy of antimalarial drugs is increasingly challenged by the emergence and spread of artemisinin partial resistance across Africa, particularly in East Africa (Kayiba et al., 2021; Rosenthal et al., 2024a; Rosenthal et al., 2024b). To anticipate and detect resistance to ACT, the WHO recommends conducting therapeutic efficacy studies (TES) every two years in each endemic country, alongside routine monitoring of molecular markers of resistance (Report on antimalarial drug efficacy, 2020). The key molecular markers validated by WHO are *Plasmodium falciparum Kelch13 (PfKelch13), Plasmodium falciparum chloroquine resistance transporter* (*Pfcrt*), *Plasmodium falciparum multidrug resistance 1* (*Pfmdr1*), *Plasmodium falciparum dihydrofolate reductase* (*Pfdhfr*), and *Plasmodium falciparum dihydropteroate synthase* (*Pfdhps*) (Compendium of molecular markers, 2025).

The molecular marker of antimalarial drug resistance, *PfKelch13,* is associated with artemisinin partial resistance (WHO Artemisinin partial resistance). The WHO has identified several mutations within the *PfKelch13* propeller domain as validated, candidates or potential markers of partial artemisinin resistance, based on evidence from laboratory, clinical, and genetic epidemiology data. Currently, 14 *PfKelch13* mutations are classified as validated, 6 as candidate, and 15 as potential markers of artemisinin partial resistance. (Compendium of molecular markers, 2025) (Supplementary Table 1).

Resistance to partner drugs includes the genes *Pfcrt*, *Pfmdr1*, *Pfdhfr*, and *Pfdhps*. Mutations in the *Pfcrt* gene are associated with chloroquine resistance, particularly those occurring at codons 72–76 (Fidock et al., 2000; Djimdé et al., 2001; Wootton et al., 2002; Awasthi and Das, 2013). Mutations in the *Pfmdr1* gene (especially at codons N86**Y**, Y184**F**, S1034**C**, N1042**D**, and D1246**Y**) as well as copy number variations, are known to modulate parasite susceptibility to multiple antimalarials, including chloroquine, lumefantrine, amodiaquine, and mefloquine (Sidhu et al., 2005; Sidhu et al., 2006; Humphreys et al., 2007; Report on antimalarial drug efficacy, 2020). Furthermore, mutations in the *Pfdhfr* gene, notably N51**I**, C59**R**, S108**N**, and I164**L**, are associated with pyrimethamine resistance. Similarly, mutations in the *Pfdhps* gene, such as S436 **A**/**F**, A437**G**, K540**E**, and A581**G**, confer resistance to sulfadoxine (Report on antimalarial drug efficacy, 2020).

In Benin, only two TES evaluating AL were published between 2010 and 2024: one conducted in 2014 in Djougou and Cobly (northern Benin) (Ogouyèmi-Hounto et al., 2016), and another in 2019 in Bohicon and Kandi (northern and central Benin) (Kpemasse et al., 2021), neither of which reported ACT treatment failure rates exceeding 10% (Ogouyèmi-Hounto et al., 2016; Kpemasse et al., 2021). Furthermore, the most recent published molecular surveillance data were based on samples collected in 2019 in Tanguiéta, in the north (L'Episcopia et al., 2023) highlighting the lack of recent and geographically representative resistance data in Benin. In the context of limited resources for routine TES, surveillance of molecular markers could serve as a proxy indicator of *P. falciparum* resistance to antimalarial drugs. Therefore, the present study aimed to assess trends in the prevalence of established antimalarial drug resistance genotypes in the *Pfkelch13*, *Pfcrt*, *Pfmdr1*, *Pfdhfr*, and *Pfdhps* genes using samples collected at two time points, 2017 and 2024, in southern Benin.

## 2 Materials and methods

### 2.1 Study sites

**Fig. 1:**
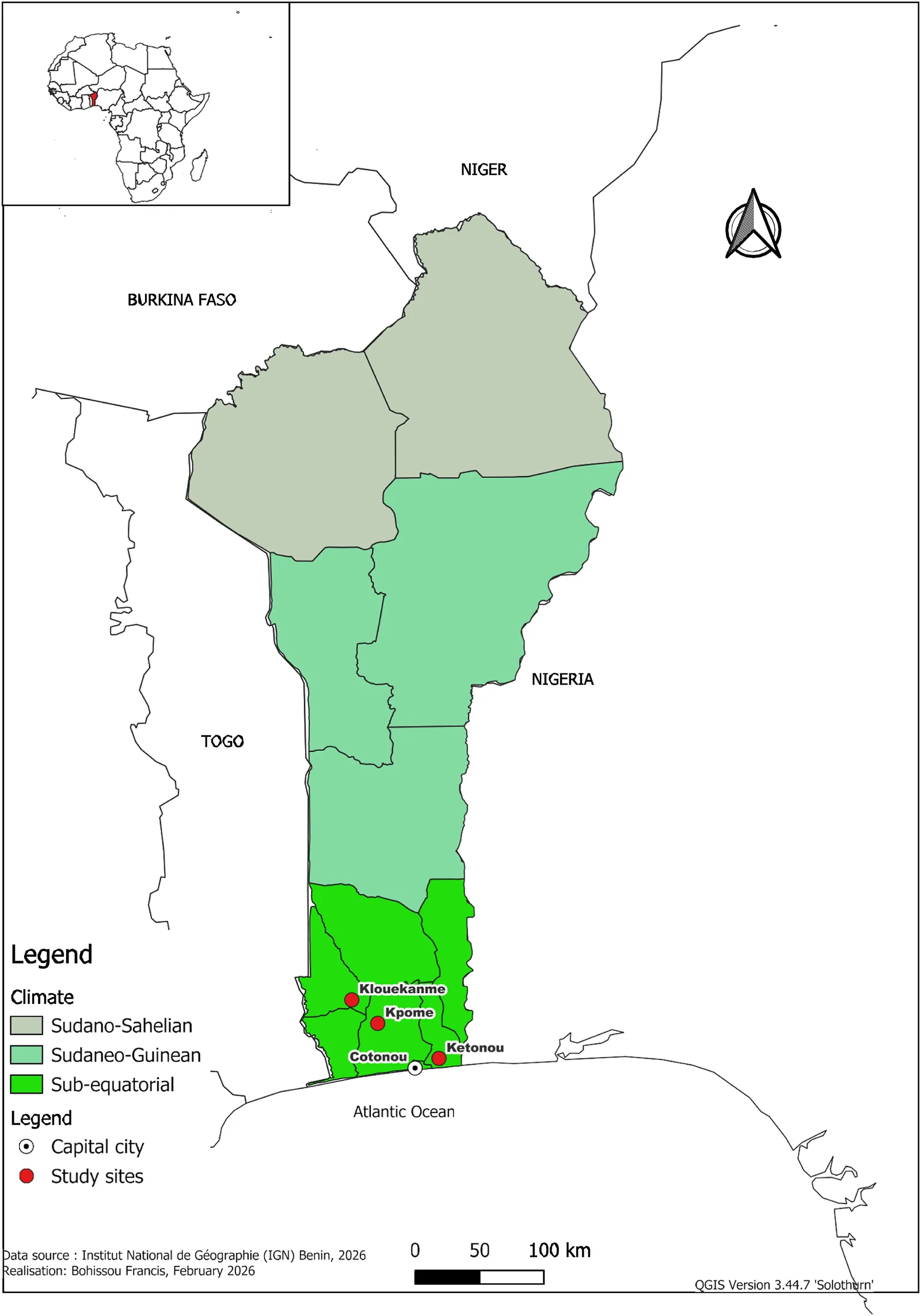
Mapping of study sites.

The studies were conducted in southern Benin, specifically in the districts of Kétonou, Kpomè, and Klouékanmè (Fig. 1). The southern region is characterized by an equatorial climate with high humidity, alternating dry seasons (November to March and mid-July to mid-September) and rainy seasons (April to mid-July and mid-September to October) (Chabi et al., 2025). Malaria transmission is perennial, with a peak during the rainy season. During the demographic survey organised in the country in 2017-2018, malaria in the southern region was meso-endemic, with an estimated prevalence of 26-34% (Statistiques Sociales Benin, 2023). The incidence of malaria in southern Benin decreased from 17% in 2017 (Hounnakan et al., 2018) to 10% in 2024 (Chabi et al., 2025).

### 2.2 Samples analysed and study population

Samples were derived from two community-based cross-sectional surveys conducted in southern Benin during the peak malaria transmission season (July–August): the first in 2017 in Kétonou and Kpomè, and the second in 2024 in Klouékanmè. The 2017 survey aimed to assess the impact of insecticide resistance on malaria prevalence and enrolled 500 children aged 6 months to 10 years at each site. The 2024 survey also included 500 children aged 3–14 years and investigated coinfection with malaria and urinary schistosomiasis. In both studies, socio-demographic and clinical data were collected. *Plasmodium* infection was diagnosed using rapid diagnostic tests (RDTs) and light microscopy (thick and thin blood smears). Dried blood spots (DBS) were collected on filter paper for molecular analysis and stored at room temperature until DNA extraction.

### 2.3 Ethical clearance

Ethical approval for these studies was obtained from the Comité d’Ethique de la Recherche de l’Institut des Sciences Biomédicales Appliquées (CER-ISBA) under reference numbers 81/CER-ISBA (04/02/2016) and 197/CER-ISBA (05/04/2024). The present molecular analysis was additionally approved by the Ethics Committee of the Faculty of Medicine, University of Tübingen, Germany (Project No. 704/2022BO2). Written informed consent was obtained from parents or guardians of enrolled children. Assent was also obtained from children aged 8 years and older. For this molecular analysis on antimalarial drug resistance, only dried blood spot samples from children with microscopy-confirmed parasitaemia were included.

### 2.4 DNA extraction and molecular analysis

DNA was extracted at the Institute of Tropical Medicine in Tübingen (Germany) from DBS (small punches 3-6 mm in diameter) using the QIAamp® Blood mini kit (Qiagen GmbH, Hilden, GERMANY) per the manufacturer's recommendations. Extracted DNA was stored at − 20°C until further use.

Sanger sequencing was performed to analyse the *PfKelch13*, *Pfmdr1*, *Pfdhfr*, and *Pfdhps* genes using previously published primers and PCR protocols targeting fragments (Ariey et al., 2014; Li et al., 2014; Bohissou et al., 2024) containing mutations known to be associated with antimalarial drug resistance. For *PfKelch13*, the propeller domain was amplified by nested PCR to generate an 849 bp fragment spanning codons 433–700. The *Pfmdr1* gene was amplified into two fragments by conventional nested PCR, with the first fragment covering codons 86 and 184 and the second covering codons 1034, 1042, and 1246. Similarly, *Pfdhfr* was amplified by nested PCR to include codons 51, 59, 108, and 164, while *Pfdhps* was amplified in two separate fragments encompassing codons 431, 436, and 437 in the first fragment and codons 540, 581, and 613 in the second. PCR products were visualised using the QIAxcel system, purified with ExoSAP-IT (Thermo Fisher Scientific) to remove residual primers and dNTPs, and submitted to Eurofins Genomics for Sanger sequencing. Sequence analysis was performed using Geneious software (version 2025.2.2), with alignment against the *P*. *falciparum* 3D7 reference sequences for *PfKelch13* (PF3D7_1343700), *Pfmdr1* (PF3D7_0709000), *Pfdhfr* (PF3D7_0417200), and *Pfdhps* (PF3D7_0810800). Mutations were identified by examining nucleotide alignment mismatches at specific codon positions within the gene. Mixed infections were inferred when sequencing chromatograms displayed clear dual peaks of comparable intensity at the locus of interest. Samples showing only minor secondary peaks were not classified as mixed infections, as low-intensity signals may reflect background noise or sequencing artefacts and cannot be reliably distinguished from low-frequency variants using Sanger sequencing. The different points of mutation at each locus analysed for the *Pfmdr1*, *Pfdhfr*, and *Pfdhps* genes associated with antimalarial resistance are summarised in Supplementary Table 2. For *PfKelch13* sequence analysis, only nucleotide positions corresponding to codons 438–691 were evaluated. Chromatograms were carefully inspected, and variants located in low-quality regions near the forward and reverse primer-binding sites were excluded to minimize the risk of including background noise or sequencing artefacts. Only mutations supported by clear and high-quality sequence signals were retained for analysis. In addition, for *PfKelch13*, nucleotide sequences were translated into amino acid sequences and subsequently aligned to identify and characterize mutations at specific codon positions.

The *Pfcrt* gene was genotyped by quantitative PCR (qPCR) using previously published primers and probes to distinguish the CVMNK, CV**IET**, and **S**VMN**T** haplotypes at codons 72–76 (Bushman et al., 2016; Nguyen et al., 2019). Prior to qPCR, samples were pre-amplified by conventional PCR. All reactions were performed in triplicate. Genomic DNA from the *P*. *falciparum* 3D7 strain served as a positive control for the chloroquine-sensitive CVMNK haplotype. In contrast, Dd2 and 7G8 strains were used as positive controls for the resistant CV**IET** and **S**VMN**T** haplotypes, respectively. DNA extracted from the whole blood of a malaria-naïve individual and nuclease-free water were included in each run as negative (non-template) controls. All qPCR assays were conducted on a LightCycler 480 Instrument II (Roche). Quantification cycle (Cq) values were determined using the second-derivative maximum method implemented in the LightCycler 480 software (version 1.5.1.62). A genotype was assigned when at least two of the three replicates yielded concordant results (the difference in Cq values did not exceed two cycles). When all three replicates produced discordant results or when amplification curves did not meet predefined quality criteria, the sample was repeated.

### 2.5 Data analysis

Descriptive analysis was performed using proportions (%) with 95% confidence intervals (CI), means, or medians, as appropriate. Parasite density obtained from microscopy readings was summarised as the geometric mean with corresponding 95% CI. We analysed *Pfdhfr* (N51**I**, C59**R**, S108**N**, and I164**L**), *and Pfdhps* (I431**V**, S436 **A/F**, A437**G**, K540**E**, A581**G**, and A613 **S/T**), together with their respective haplotypes, including the combined two-locus *Pfdhfr–Pfdhps* haplotypes. We also evaluated *Pfcrt* haplotypes (CVMNK, CV**IET**, and **S**VMN**T**), *Pfmdr1* mutations (N86**Y**, Y184**F**, S1034**C**, N1042**D**, and D1246**Y**) and their corresponding variant combinations, as well as synonymous and non-synonymous mutations in *PfKelch13*.

For each gene, the prevalence of alleles was reported as wild type (only wild-type allele detected), mutant (only mutant allele detected), or mixed infection (both wild-type and mutant alleles detected). In variant combination analysis, mixed infections were classified as mutants. Two-proportion tests (Chi-square test or Fisher's exact test when appropriate) were used to assess associations in the prevalence of drug resistance markers between the two survey periods (2017 vs 2024). The Wilcoxon rank-sum test and the Kruskal-Wallis test were used to compare the mean and median. Bonferroni correction was applied for multiple comparisons. The software R (RStudio version 4.3.1; R Core Team (2023) from R Foundation for Statistical Computing, Vienna, Austria < https://www.R-project.org/>) was used for statistical analysis. All statistical analysis were performed at the 5% significance level. GraphPad Prism 11.0 (GraphPad Software, San Diego, California, USA) was used to generate the graphs.

## 3 Results

### 3.1 Population characteristics

A total of 358 participants with microscopy-confirmed *P. falciparum* malaria were included in the analysis, comprising 69 cases in 2017 and 289 in 2024. The median age was 7 years (IQR 5–10), with 28% (96/345) aged ≤5 years. The gender distribution was balanced. The median axillary temperature at presentation was 36.8°C, and 9.8% (35/358) of patients were febrile (≥37.5°C), indicating that the study population was predominantly asymptomatic. The geometric mean parasite density (GMPD) was 461 parasites/μL (95% CI: 365–581; n = 358). Detailed population characteristics stratified by year are presented in Table 1.

**Table 1:** Baseline characteristics of the study population included in 2017 (N = 69) and 2024 (N = 289) in Benin.

Variables	N	2017	2024
**Sites (Southern Benin)**	358	N = 69	N = 289
Ketonou		24 (34.8%)	0 (0%)
Klouekanme		0 (0%)	289 (100%)
Kpome		45 (65.2%)	0 (0%)
**Sex**	358		
Female		32 (46.4%)	145 (50.2%)
Male		37 (53.6%)	144 (49.8%)
**Age (Median [IQR])**	358	5.0 [3.0, 8.0]	8.0 [6.0, 10.0]
**Age group**	358		
≤5 years		35 (50.7%)	69 (23.9%)
>5 years		34 (49.3%)	220 (76.1%)
**Temperature (Median [IQR])**	358	36.6 [36.3, 37.1]	36.8 [36.4, 37.1]
**Fever (Temperature ≥37.5°C)**	358	10 (14.5%)	25 (8.7%)
**GMPD (95%CI)**	358	1076 [679, 1706]	350 [270, 454]

*GMPD: Geometric mean of parasitemia density, IQR: Interquartile range, CI: Confidence interval.

### 3.2 Polymorphisms in antimalarial resistance markers

Molecular marker genotyping was successful in 78.5% (281/358) of samples for *PfKelch13*, 98.3% (352/358) for *Pfcrt,* 94.4% (338/358) for *Pfmdr1* (both part 1 and part 2), 96.1% (344/358) for *Pfdhfr,* and 81.3% (291/358) for both part 1 and part 2 of *Pfdhps* (Supplementary Table 3).

***PfKelch13*:** Non-synonymous mutations were detected in 2.5% (7/281) of successfully sequenced isolates. Greater genetic diversity was observed in 2024, with six distinct non-synonymous mutations identified at different codon positions (F491**L**, I540**V**, A578**S**, E602**G**, A621**S**, and A676**S**). None of the detected mutations in *PfKelch13* corresponded to validated, candidate, or potential markers associated with artemisinin partial resistance (Table 2, Supplementary Tables 4 and 5).

**Table 2:** Frequency of *PfKelch13* genes in *P. falciparum* isolates from Benin in 2017 (N = 25) and 2024 (N = 256). Bold letters indicate a mutant amino acid.

*PfKelch13*	Mutation	2017	2024
**Non - synonymous**	A578**S**	0 (0%)	1 (0.4%)
	A621**S**	0 (0%)	1 (0.4%)
	A676**S**	0 (0%)	1 (0.4%)
	E602**G**	0 (0%)	1 (0.4%)
	F491**L**	0 (0%)	1 (0.4%)
	I540**V**	0 (0%)	1 (0.4%)
	Q613**H**	1 (4.0%)	0 (0%)
**Wild Type**	Wild Type	23 (92%)	241 (94.0%)

**Fig. 2:**
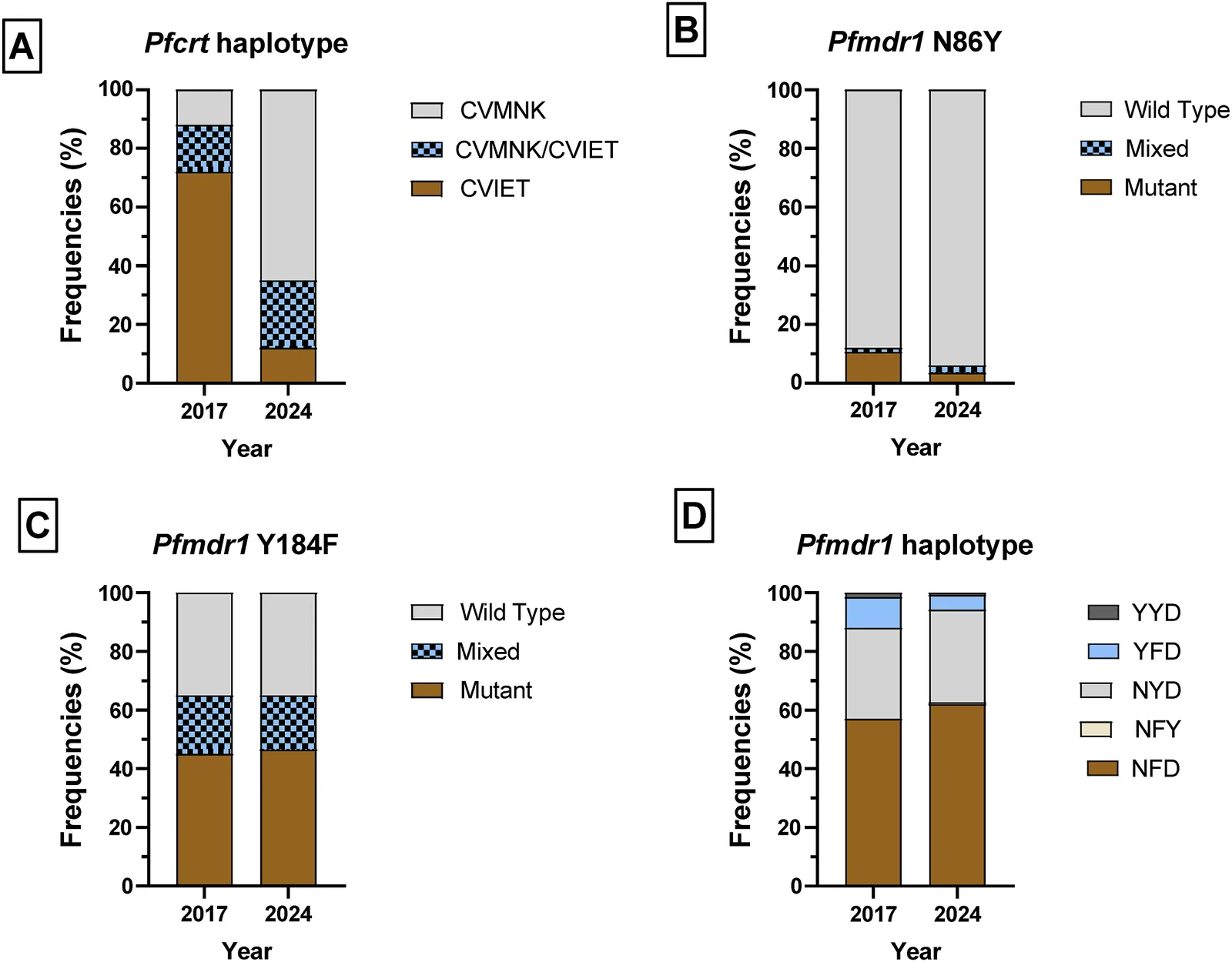
Evolution of the prevalence of mutant, mixed and wild-type alleles of *Pfcrt* and *Pfmdr1* between 2017 and 2024 in southern Benin. **(A)***Pfcrt* (2017: N = 67; 2024: N = 285); **(B)***Pfmdr1* N86**Y***and***(C)***Pfmdr1* Y184**F** (2017: N = 69; 2024: N = 285); **(D)***Pfmdr1* haplotype (2017: N = 65; 2024: N = 273).

***Pfcrt-Pfmdr1***: The combined prevalence of the mutant *Pfcrt* haplotype (CV**IET**) and mixed infections (CV**IET**/CVMNK) declined markedly from 88.1% (59/67; 95% CI: 78.2–93.8%) in 2017 to 35.4% (101/285; 95% CI: 30.0–41.1%) in 2024 (Chi-square test = 60.58, p < 0.001) (Fig. 2A; Supplementary Table 6). Similarly, the combined proportion of mixed and mutant isolates at *Pfmdr1* N86**Y** decreased from 11.6% (8/69; 95% CI: 6.0–21.1%) in 2017 to 6.0% (17/285; 95% CI: 3.8–9.4%) in 2024, although this reduction was not statistically significant (Fisher test, p = 0.116) (Fig. 2B; Supplementary Table 6). In contrast, the prevalence of *Pfmdr1* Y184**F** (mixed and mutant combined) remained stable between 2017 (65.2%; 45/69; 95% CI: 53.4–75.4%) and 2024 (65.3%; 186/285; 95% CI: 59.6–70.6%) (Fig. 2C; Supplementary Table 6). No mutations were detected at *Pfmdr1* S1034**C** or N1042**D**, and only one mixed infection (0.4%; 95% CI: 0.06–2.04%) at D1246**Y** (N = 275) was identified in 2024. Overall, there were no statistically significant differences in the distribution of *Pfmdr1* variant combinations between 2017 and 2024 (Fisher test, p = 0.379). However, a slight increase was observed in the predominant *Pfmdr1* N**F**D (N86-184**F**-D1246) variant combination, rising from 56.9% (37/65; 95% CI: 44.8–68.2%) in 2017 to 61.9% (169/273; 95% CI: 56.0–67.4%) in 2024 (Fig. 2D; Supplementary Table 6).

**Fig. 3:**
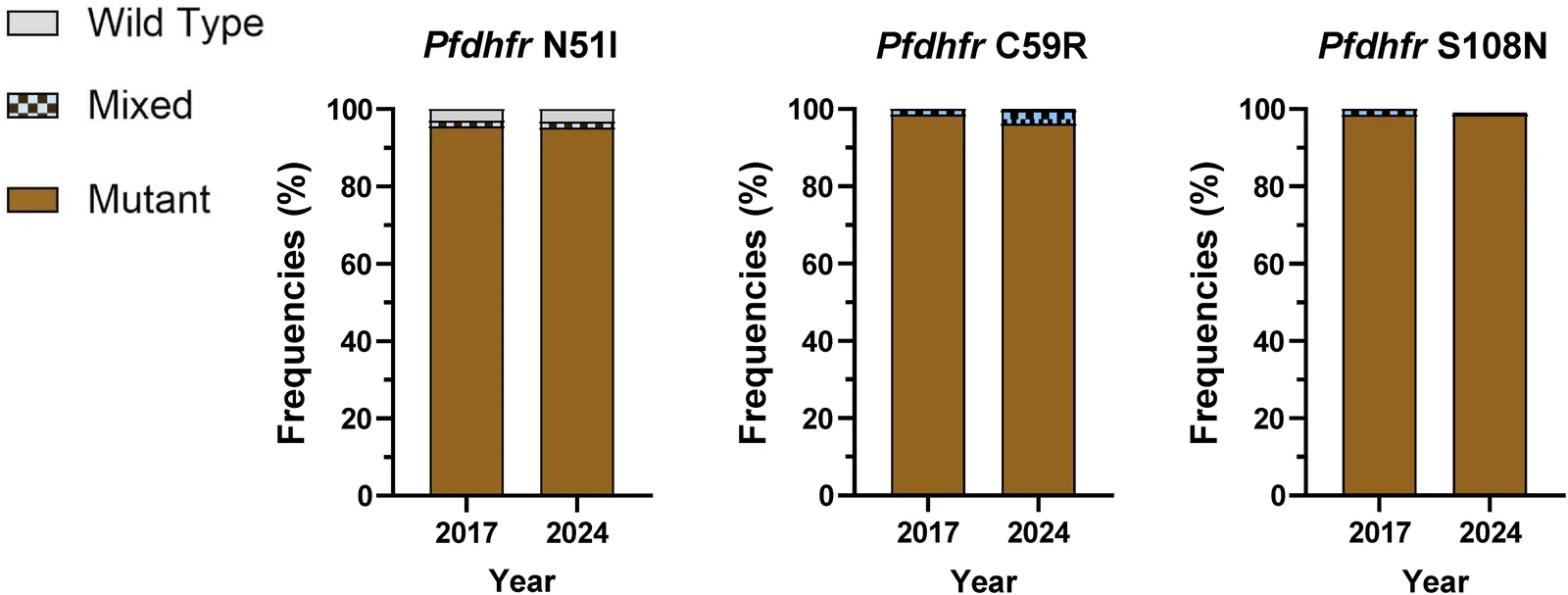
Evolution of the prevalence of mutant, mixed and wild-type alleles of *Pfdhfr* between 2017 and 2024 in southern Benin. *Pfdhfr* codons (2017: N = 66; 2024: N = 278).

***Pfdhfr/Pfdhps:*** No mutations were detected at *Pfdhfr* I164**L**. In contrast, mutations at codons *Pfdhfr* N51**I**, C59**R**, and S108**N** were nearly fixed, with prevalences ranging from 96.8% to 100% (Fig. 3; Supplementary Table 7) over time. Accordingly, the triple-mutant *Pfdhfr* variant combination **IRN**I was almost fixed, accounting for 97.0% (64/66; 95% CI: 89.6–99.2%) in 2017 and 96.4% (268/278; 95% CI: 93.5–98.0%) in 2024 (Table 3).

**Table 3:** Frequency of *Pfdhfr*, *Pfdhps*, and *Pfdhfr/Pfdhps* genes in *P. falciparum* isolates from Benin in 2017 and 2024. Bold letters indicate a mutant amino acid.

Variant combination	Type	2017	2024
***Pfdhfr***		N = 66	N = 278
N_51_C_59_S_108_I_164_	Wild Type	0 (0%)	0 (0%)
**I**C**N**I	Double	0 (0%)	1 (0.4%)
N**RN**I	Double	2 (3.0%)	9 (3.2%)
**IRN**I	Triple	64 (97.0%)	268 (96.4%)
***Pfdhps***		N = 60	N = 231
I_431_S_436_A_437_K_540_A_581_A_613_	Wild Type	0 (0%)	1 (0.4%)
I**A**AKAA	Single	1 (1.7%)	3 (1.3%)
IS**G**KAA	Single	41 (68.3%)	132 (57.1%)
I**AG**KAA	Double	11 (18.3%)	49 (21.2%)
I**FG**KAA	Double	0 (0%)	2 (0.9%)
IS**G**KA**S**	Double	1 (1.7%)	2 (0.9%)
IS**G**K**G**A	Double	0 (0%)	3 (1.3%)
**V**S**G**KAA	Double	0 (0%)	4 (1.7%)
I**AG**KA**S**	Triple	1 (1.7%)	8 (3.6%)
I**AG**K**G**A	Triple	0 (0%)	3 (1.3%)
I**FG**KA**S**	Triple	0 (0%)	1 (0.4%)
IS**G**K**GS**	Triple	0 (0%)	1 (0.4%)
**VAG**KAA	Triple	0 (0%)	10 (4.3%)
**V**S**G**K**G**A	Triple	0 (0%)	1 (0.4%)
I**AG**K**GS**	Quadruple	0 (0%)	2 (0.9%)
**VAG**KA**S**	Quadruple	0 (0%)	1 (0.4%)
**VAG**K**G**A	Quadruple	0 (0%)	3 (1.3%)
**VAG**K**GS**	Quintuple	5 (8.3%)	5 (2.2%)
**Combined *Pfdhfr/Pfdhps***		N = 57	N = 224
N_51_C_59_S_108_I_164_/I_431_S_436_A_437_K_540_A_581_A_613_	Wild Type	0 (0%)	0 (0%)
**IRN**I/ISAKAA	Triple	0 (0%)	1 (0.4%)
N**RN**I/IS**G**KAA	Triple	1 (1.7%)	7 (3.1%)
**I**C**N**I/I**AG**KAA	Quadruple	0 (0%)	1 (0.4%)
**IRN**I/I**A**AKAA	Quadruple	1 (1.7%)	3 (1.3%)
**IRN**I/IS**G**KAA	Quadruple	37 (64.9%)	120 (53.6%)
**IRN**I/I**AG**KAA	Quintuple	11 (19.4%)	47 (21.0%)
**IRN**I/I**FG**KAA	Quintuple	0 (0%)	2 (0.9%)
**IRN**I/IS**G**KA**S**	Quintuple	1 (1.7%)	2 (0.9%)
**IRN**I/IS**G**K**G**A	Quintuple	0 (0%)	2 (0.9%)
**IRN**I/**V**S**G**KAA	Quintuple	0 (0%)	4 (1.8%)
**IRN**I/I**AG**KA**S**	Sextuple	1 (1.7%)	8 (3.6%)
**IRN**I/I**AG**K**G**A	Sextuple	0 (0%)	3 (1.3%)
**IRN**I/I**FG**KA**S**	Sextuple	0 (0%)	1 (0.4%)
**IRN**I/IS**G**K**GS**	Sextuple	0 (0%)	1 (0.4%)
**IRN**I/**VAG**KAA	Sextuple	0 (0%)	10 (4.5%)
**IRN**I/**V**S**G**K**G**A	Sextuple	0 (0%)	1 (0.4%)
**IRN**I/I**AG**K**GS**	Septuple	0 (0%)	2 (0.9%)
**IRN**I/**VAG**KA**S**	Septuple	0 (0%)	1 (0.4%)
**IRN**I/**VAG**K**G**A	Septuple	0 (0%)	3 (1.3%)
**IRN**I/**VAG**K**GS**	Octuple	5 (8.9%)	5 (2.2%)

No mutation was observed at *Pfdhps* K540**E**. The *Pfdhps* A437**G** mutation remained fixed over time, with a combined prevalence of mutant and mixed infections of approximately 98% at both time points. The prevalence of mutant and mixed genotypes at *Pfdhps* I431**V** (reported in Nigeria) and *Pfdhps* S436 **A/F** increased slightly between 2017 and 2024, from 8.3% (5/60; 95% CI: 3.6–18.1%) to 10.5% (29/275; 95% CI: 7.4–14.8%) for *Pfdhps* I431**V** and from 28.3% (17/60) to 34.5% (92/275; 95% CI: 28.2–39.3%) for *Pfdhps* S436 **A/F**; however, these changes were not statistically significant (Chi-square test = 0.264, p = 0.607 and Chi-square test = 0.854, p = 0.355, respectively).

**Fig. 4:**
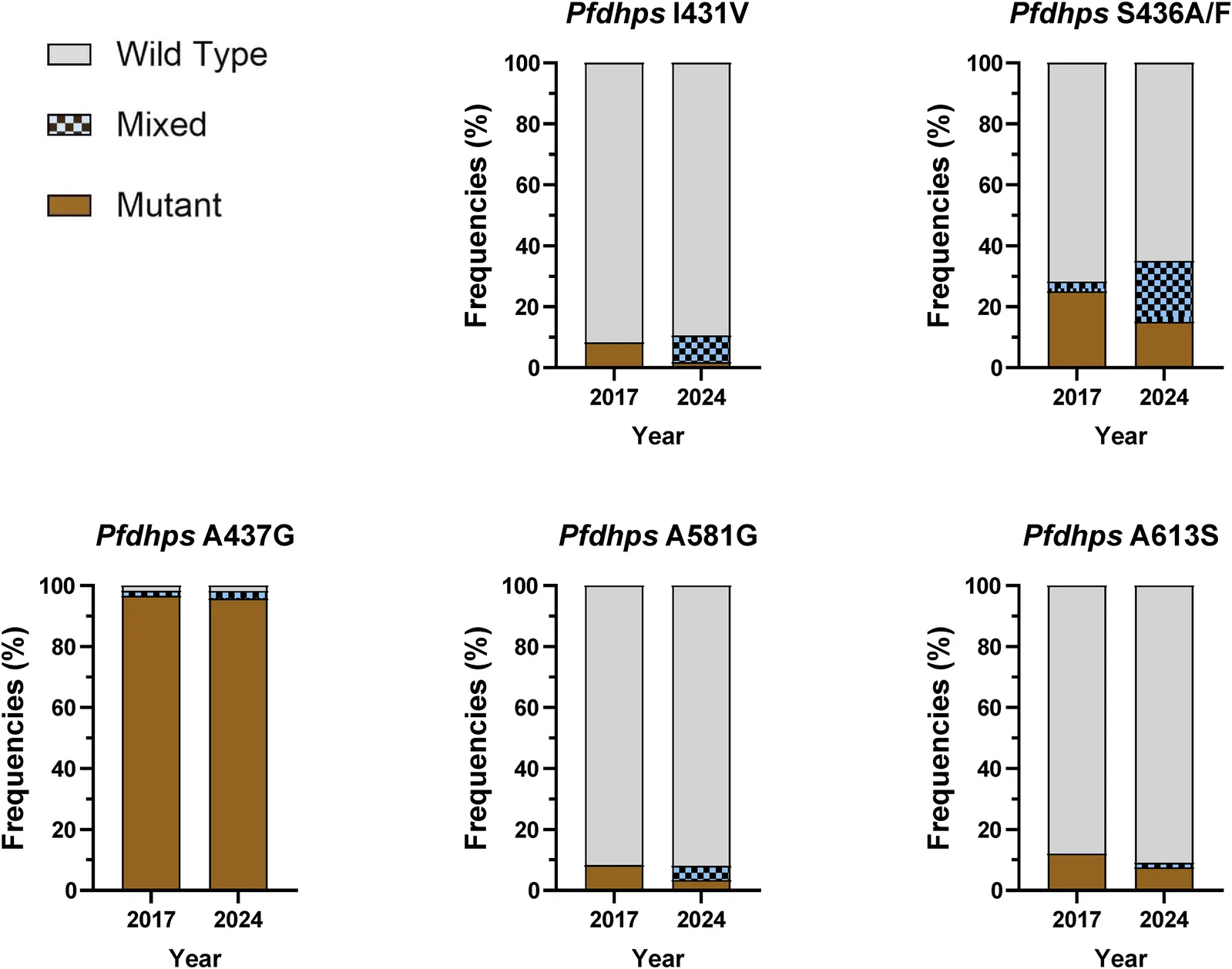
Evolution of the prevalence of mutant, mixed and wild-type alleles of *Pfdhps* between 2017 and 2024 in southern Benin. (a) *Pfdhps* codons (2017: N = 60; 2024: N = 275)*; (b) Pfdhps* codons (2017: N = 60; 2024: N = 240).

Conversely, the combined prevalence of mutant and mixed infections at *Pfdhps* A581**G** and A613**S** showed a slight decline between 2017 and 2024, from 8.3% (5/60; 95% CI: 3.6–18.1%) to 7.9% (19/240; 95% CI: 5.1–12.0%) for A581**G** and from 11.7% (7/60; 95% CI: 5.8–22.2%) to 8.8% (21/240; 95% CI: 5.8–13.1%) for A613**S**, with no statistically significant differences (Fisher test, p = 1 and Chi-square test = 0.483, p = 0.487, respectively). Notably, all five isolates carrying *Pfdhps* I431**V** and *Pfdhps* A581**G** mutations in 2017 were identified in Ketonou (N = 22), a study site located approximately 20 km from the Nigerian border (Fig. 4; Supplementary Table 7).

Greater *Pfdhps* variant combination diversity was observed in 2024 (n = 18) compared with 2017 (n = 6). The most prevalent *Pfdhps* variant combination was IS**G**KAA, characterized by a single mutation at codon A437**G**. Its prevalence declined from 68.3% (41/60; 95% CI: 55.8–78.6%) in 2017 to 57.1% (132/231; 95% CI: 50.6–63.4%) in 2024. The double-mutant variant combination **AG**KAA increased slightly from 18.3% (11/60; 95% CI: 10.5–30.1%) in 2017 to 21.2% (49/231; 95% CI: 16.4–26.9%) in 2024. The quintuple-mutant variant combination **VAG**K**GS** was detected at 8.3% (5/60; 95% CI: 3.6–18.1%) in 2017 and 2.2% (5/231; 95% CI: 0.9–4.9%) in 2024. None of these differences was statistically significant (Table 3).

Analysis of the combined *Pfdhfr/Pfdhps* genotypes revealed that the triple *Pfdhfr* **IRN** mutation associated with a single mutation at *Pfdhps* 437**G** (**IRN**_**G**), linked to early resistance to sulfadoxine–pyrimethamine, was highly prevalent in both years. The prevalence was 96.5% (55/57; 95% CI: 88.1–99.0%) in 2017 and 94.6% (212/224; 95% CI: 90.8–96.9%) in 2024, with no statistically significant difference between the two time points (p = 0.7424). The quintuple mutation associated with the triple mutation *Pfdhfr* **IRN** and double mutation *Pfdhps* A437**G** and A581**G** (**IRN**_**GG**) was 8.8% in (5/57; 95% CI: 3.8–18.9%) in 2017 and 6.7% (15/224; 95% CI: 4.1–10.7%) in 2024, with no statistical difference (Chi-square test = 0.296, p = 0.5689) (Table 3).

### 3.3 Comparison of parasitemia and fever status between variants

**Fig. 5:**
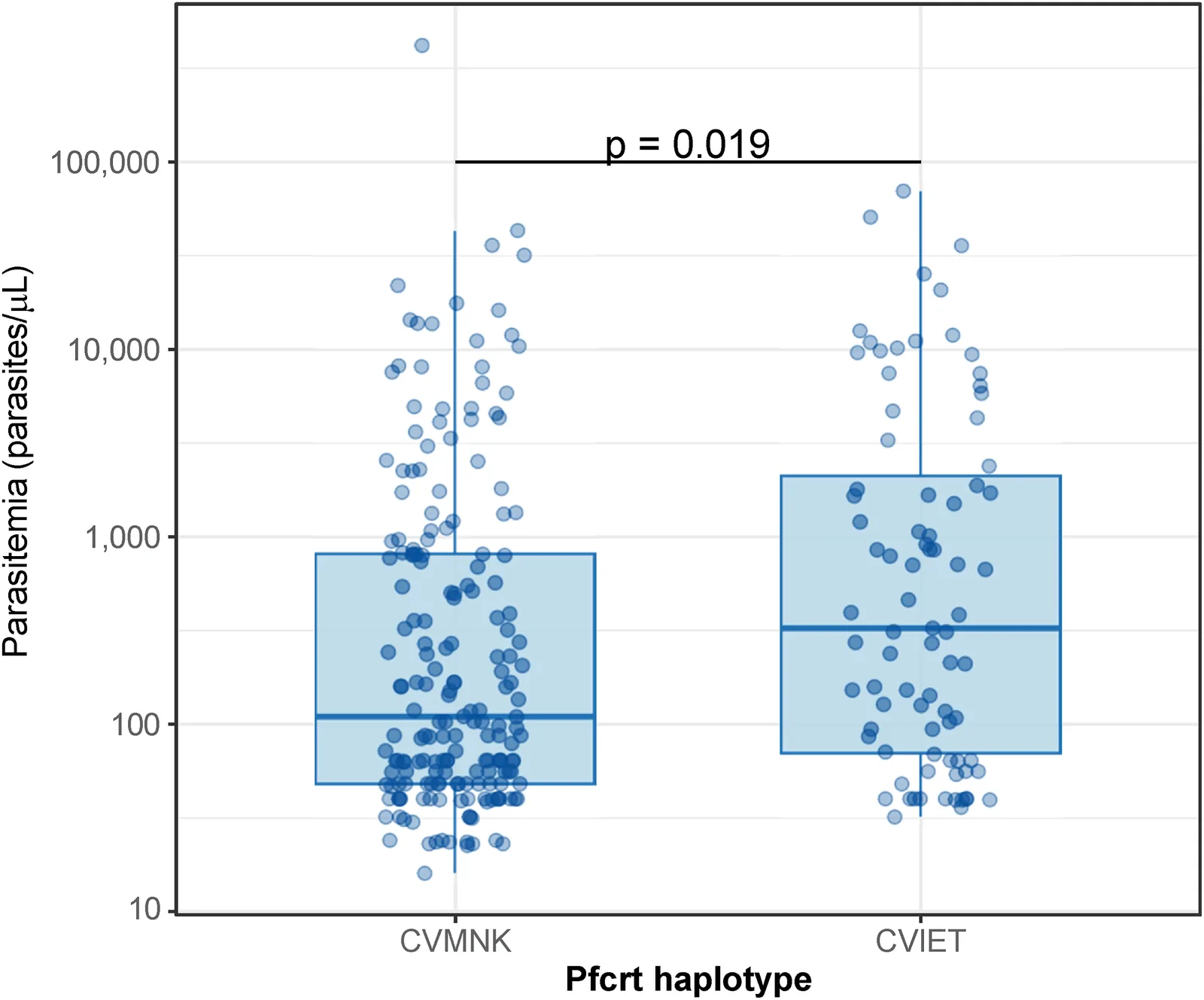
Comparison of parasitemia between the resistant *Pfcrt* variant CV**IET** (N = 83) and the wild type *Pfcrt* variant CVMNK (N = 192) in samples collected in 2017 and 2024 in Southern Benin.

**Parasitemia**: The geometric mean parasitemia did not differ significantly between *PfKelch13* mutant and wild type isolates (359.3 vs 315.2 parasites/μL; Wilcoxon rank-sum test, p = 0.939). Similarly, no significant differences were observed between *Pfmdr1* variants (N**F**D: 344.3 vs NYD: 254.1 parasites/μL; Wilcoxon rank-sum test, p = 0.175) or among the combined *Pfdhfr*/*Pfdhps* haplotypes (Kruskal–Wallis test, p = 0.125) (Supplementary Table 8). In contrast, isolates carrying the chloroquine-resistant *Pfcrt* CV**IET** variant had a significantly higher geometric mean parasitemia than those with the wild type CVMNK variant (516.2 vs 241.8 parasites/μL; Wilcoxon rank-sum test with Bonferroni correction, p = 0.019) (Fig. 5 and Supplementary Table 8).

**Fever**: The occurrence of fever did not differ significantly according to any of the molecular marker groups analysed (Supplementary Table 9). Fever was observed in 11.8% (2/17) of isolates carrying *PfKelch13* mutations compared with 9.8% (26/264) of wild type isolates (Fisher test, p = 0.681). Similarly, no significant differences were found between *Pfcrt* variants (CV**IET**: 13.3% vs CVMNK: 9.4%; Fisher test, p = 0.393), *Pfmdr1* variants (N**F**D: 11.7% vs NYD: 7.5%; Fisher test, p = 0.326), or among the combined *Pfdhfr/Pfdhps* variants (Fisher test, p = 0.915).

## 4 Discussion

This study provides updated evidence on the temporal changes in *P*. *falciparum* molecular markers associated with antimalarial drug resistance in southern Benin between 2017 and 2024. The marked decline in the *Pfcrt* CV**IET** haplotype (88.1% vs 35.4%) and the *Pfmdr1* N86**Y** mutation (11.6% vs 6.0%) between 2017 and 2024 is consistent with the progressive return of chloroquine-sensitive parasites following chloroquine withdrawal, a trend previously documented in Malawi (Kublin et al., 2003; Laufer et al., 2010) and subsequently across sub-Saharan Africa (Lucchi et al., 2015; Balikagala et al., 2020; Amusan et al., 2024; Millogo et al., 2025; Somé et al., n.d.; Juliano et al., 2024; Von Wowern et al., 2024; Wernsman Young et al., 2025). Nevertheless, the prevalence of *Pfcrt* CV**IET** and *Pfmdr1* N86**Y** remained relatively high in southern Benin compared with previous observations from northern Benin in 2019, where the prevalence of *Pfcrt* K76**T** and *Pfmdr1* N86**Y** was 4% and 0%, respectively. (L'Episcopia et al., 2023). These differences likely reflect geographic heterogeneity between the north and the south, and were also observed by Cavros et al., who reported in 2022 prevalence estimates of *Pfcrt* CV**IET** of 29% and 2% in the south (Allada) and north (Parakou), respectively (Cavros et al., 2026). While malaria transmission is seasonal in the north and perennial in the south, these contrasting transmission patterns may influence antimalarial drug use and parasite population dynamics.

Despite its marked decline, *Pfcrt* CV**IET** prevalence remained relatively high in 2024 (35.4%), in contrast to the near disappearance of chloroquine-resistance markers reported in Malawi following chloroquine withdrawal (Kublin et al., 2003; Laufer et al., 2010). Moreover, in our study, parasites harbouring the resistant *Pfcrt* CV**IET** exhibited higher parasite densities than those carrying the wild type *Pfcrt* CVMNK. However, this difference was not associated with clinical manifestations, and previous studies have reported inconsistent associations between *Pfcrt* genotype and parasite density (Afoakwah et al., 2014; Ehrhardt et al., 2007; Hassen et al., 2022). ASAQ is part of the CTA used for uncomplicated malaria in Benin. The continued use of amodiaquine, which shares structural similarities with chloroquine and overlapping resistance mechanisms, may have contributed to the persistence of *Pfcrt* resistance-associated alleles in the parasite population. However, further studies are needed to clarify the factors maintaining these resistant alleles.

Furthermore, the sustained high prevalence (60% in 2024) of the *Pfmdr1* N**F**D haplotype, which is associated with reduced lumefantrine susceptibility (Bakari et al., 2024), warrants close attention, given the widespread reliance on AL. However, the TES conducted in 2019 in Benin reported AL failure rates below 10%, with PCR-corrected failure estimated at 3.7% and 3.3% in Bohicon (Central part) and Kandi (northern part), respectively (Kpemasse et al., 2021). Similarly, the 2022 TES showed AL failure rates of 2.7%, 1.6%, and 5.5% in Allada (southern part), Bohicon, and Parakou (northern part), respectively (Cavros et al., 2026). Reassuringly, none of the non-synonymous *PfKelch13* mutations detected in our study is currently classified by the WHO as validated, associated, or potential markers of artemisinin partial resistance, consistent with findings from Benin and across West Africa (Bazie et al., 2020; Ndwiga et al., 2021; World Malaria Report, 2025).

In contrast, artemisinin partial resistance has been documented in East Africa, notably in Rwanda, Uganda, and Ethiopia (Kayiba et al., 2021; Ndwiga et al., 2021; Gashema et al., 2025; World Malaria Report, 2025). The mutation A578**S**, found in our study and previously reported in Benin (Ménard et al., 2016) and widely documented across Africa and Asia (Ménard et al., 2016; WHO Artemisinin partial resistance; Kamau et al., 2015; Aninagyei et al., 2020; Ajogbasile et al., 2022; Kayiba et al., 2021; Ndwiga et al., 2021; Arzika et al., 2023; Martín Ramírez et al., 2025) has not been associated with delayed parasite clearance or increased activity in the ring-stage survival assay *in vitro* (Kamau et al., 2015; Taylor et al., 2015). Other variants detected in our study have also been reported in Africa, including A621**S** (Ikeda et al., 2018; Gohoun et al., 2025), A676**S** (Jin et al., 2021), and Q613**H** (Ajogbasile et al., 2022) sporadically or at low frequencies, with no evidence of an increasing trend over time (Kayiba et al., 2021; Oyegbade et al., 2025).

The continuous detection of novel *PfKelch13* variants across sub-Saharan Africa (Kayiba et al., 2021; Oyegbade et al., 2025) likely reflects the intensive molecular surveillance currently focused on this gene. Moreover, our study reported an increase in the diversity of *PfKelch13 variants* from 2017 (1 variant) to 2024 (6 variants). Similar increases in *PfKelch13* genetic diversity were observed in Southeast Asia and Rwanda before the emergence and establishment of validated artemisinin resistance mutations (Ashley et al., 2014; Uwimana et al., 2021). However, the significance of the variants identified in the present study remains unclear, as none have been reported to be associated with artemisinin partial resistance.

Our study identified a near-fixed quadruple haplotype combining the triple *Pfdhfr* **IRN** mutation with the *Pfdhps* A437**G** mutation, a molecular profile associated with early SP resistance (Naidoo and Roper, 2013) that was already reported at a similarly high prevalence in southern Benin in 2017 (Svigel et al., 2021). This finding was confirmed across several African settings, such as Nigeria (Fagbemi et al., 2020), Burkina Faso (Bohissou et al., 2024), Ghana (Dosoo et al., 2022), Gabon (Boukoumba et al., 2021), and Cameroon (L'Episcopia et al., 2021), indicating that this partially resistant genotype is now widely established across the continent (Naidoo and Roper, 2013). In contrast, lower prevalences were reported in northern Benin, including Djougou in 2017 (81.1%) Svigel et al. (2021) and Tanguiéta in 2019 (58.8%) (L'Episcopia et al., 2023), possibly reflecting variations in SP drug pressure compared with southern Benin, including greater IPTp-SP use in the more densely populated south. For instance, substantially more pregnant women received at least three doses of SP in southern than northern Benin in 2024 (231,655 vs 121,845) (Chabi et al., 2025). However, because SP is no longer used as a first-line treatment in most African countries, its clinical impact on malaria treatment is limited. Importantly, IPTp-SP generally remains effective in areas where partial resistance predominates (van Eijk et al., 2019).

In our study, no *Pfdhps* K540**E** mutation, associated with high-level SP resistance (Naidoo and Roper, 2013), was detected, while the prevalence of A581**G** remained below 10%, consistent with previous reports from Benin (Svigel et al., 2021; L'Episcopia et al., 2023) and West Africa (Amimo et al., 2020) such as Burkina Faso (Bohissou et al., 2024; Millogo et al., 2025; Zida et al., 2024), Niger (Laminou et al., 2025), Togo (Zhou et al., 2024), and Nigeria (Martín Ramírez et al., 2025; Obaniyi et al., 2025). In contrast, K540**E** is almost fixed in East Africa, including Uganda and Rwanda, while A581**G** is moderately prevalent (Amimo et al., 2020; Flegg et al., 2022), raising questions about the current efficacy of SP for malaria prevention in these settings (Eijk et al., 2025). As WHO thresholds for IPTp-SP (>95% K540**E** and >10% A581**G**) (Meeting report of the Evidence Review Group, report) and for SMC (>50% K540**E**) (Malaria guidelines, 2025) have not been reached in Benin, our findings do not support SP withdrawal but rather the importance of continued surveillance particularly following the introduction of SMC in northern Benin in 2019 (World Malaria Report, 2025).

The detection of the *Pfdhps* I431**V** mutation (first described in Nigeria in 2007 (Sutherland et al., 2009)) and its increasing prevalence are of particular concern. In Klouékanmè (southern Benin), its prevalence rose to 10.5% in 2024, compared with 4.4% in 2017 (Svigel et al., 2021). This level is also higher than that reported in northern Benin, where prevalences of 3.6% (Djougou in 2017) (Svigel et al., 2021) and 2.0% (Tanguiéta in 2019) (L'Episcopia et al., 2023) were documented. The emergence and spread of I431**V** have been reported in several West African countries, including Burkina Faso (Bohissou et al., 2024; Somé et al., n.d.), Togo (Dorkenoo et al., 2024), Niger (Laminou et al., 2024), Mali (Vanheer et al., 2023), and Ghana (Dosoo et al., 2022), suggesting regional expansion. This mutation frequently occurs in combination with S436**A**, A437**G** and A581**G**, or A613**S**, forming haplotypes such as **VAG**K**GS** or **VAG**KA**S**. Although the precise phenotypic impact of these newly identified variants remains to be fully elucidated, a study conducted in Cameroon reported detecting composite haplotypes such as C**IRN**I/**VAG**K**GS** among pregnant women exposed to IPTp-SP, compared with non-exposed women (Cohen et al., 2023). This raises concern about a potential contribution to reduced SP efficacy.

Notably, *Pfdhps* I431**V** has not been reported in East Africa (Guémas et al., 2023), where the *Pfdhps* K540**E** mutation is highly prevalent and serves as a well-established marker of high-level SP resistance. This geographic contrast suggests that I431**V** could represent a region-specific evolutionary pathway toward SP resistance in West and Central Africa, potentially playing a role analogous to that of K540**E** in East Africa (Guémas et al., 2023). In 2017, *Pfdhps* I431**V** was detected only in Ketonou, a district located near the Nigerian border, where intense population movement may facilitate cross-border parasite flow. This pattern supports the need for coordinated, cross-border molecular surveillance, strengthened by regional networks, to monitor and contain the spread of emerging resistance markers. Further investigations are needed to clarify the impact of I431**V** on SP efficacy in both IPTp-SP and SMC.

Limitations of this study include the use of cross-sectional survey samples from only three sites in southern Benin, which may limit the generalisability of the findings to other regions of the country or to different epidemiological settings. The comparison of resistance marker prevalence across study periods should be interpreted with caution, as the study populations differed slightly in age and sampling locations. Although all sites were located in southern Benin and shared similar malaria epidemiology and control strategies. In addition, the absence of linkage to TES precludes a direct correlation between molecular findings and phenotypic resistance. In our study, we did not assess other loci associated with reduced artemisinin susceptibility beyond *PfKelch13*, including *Pfcoronin* (Demas et al., 2018), *Pfubp1*, and *Pfap2mu* (Henriques et al., 2014). This may have limited our ability to detect alternative genetic markers of artemisinin susceptibility, particularly in West Africa, where the rare validated *PfKelch13* markers (F446I, M476I, C580Y, A675V) have been reported (Oyegbade et al., 2025). However, to date, *PfKelch13* is the only marker validated by the WHO associated with artemisinin partial resistance. The relatively small sample size of the 2017 study further limits the robustness of the conclusions. Moreover, we did not analyse sub-microscopic infections because we included only microscopic-positive samples in the molecular analysis. The analysis of mixed infection as a mutation in variant combinations may have led to an overestimation of the prevalence of mutant haplotypes. Despite these limitations, this study provides s an overview of antimalarial drug resistance markers among circulating parasite populations in southern Benin and contributes to understanding local resistance patterns. However, future studies should include more study sites, larger sample sizes, and integration with TES to strengthen the evidence base.

## 5 Conclusion

Our study provides an updated overview of molecular markers associated with antimalarial drug resistance in Benin, highlighting their changes between two time points. Although there are currently no validated markers of artemisinin partial resistance that could threaten the efficacy of ACTs in the country, sustained molecular surveillance and regular TES remain essential to promptly detect any emergence or spread of resistance. The detection of specific mutations, such as *Pfdhps* I431**V**, further emphasizes the importance of establishing a coordinated regional surveillance network for monitoring and addressing antimalarial resistance.

## CRediT authorship contribution statement

**Francis Emmanuel Towanou Bohissou:** Writing – original draft, Visualization, Resources, Project administration, Methodology, Investigation, Formal analysis, Data curation, Conceptualization. **Diolinda Nahum:** Writing – review & editing, Project administration, Investigation, Data curation. **Juliana Inoue:** Writing – review & editing, Validation, Supervision, Methodology, Data curation, Conceptualization. **Paul Sondo:** Writing – review & editing, Validation, Supervision, Methodology, Data curation, Conceptualization. **Lazare Hounsou:** Writing – review & editing, Investigation, Data curation. **Gethaime Sondjo:** Writing – review & editing, Investigation, Data curation. **Nicaise Denis Codjo Djègbè:** Writing – review & editing, Investigation, Data curation. **Robinson Woli:** Writing – review & editing, Investigation, Data curation. **Salomon Dossou:** Writing – review & editing, Investigation, Data curation. **Charlotte Kpohonnou:** Writing – review & editing, Investigation, Data curation. **Halidou Tinto:** Writing – review & editing, Validation, Supervision, Resources, Project administration, Funding acquisition, Conceptualization. **Jana Held:** Writing – review & editing, Validation, Supervision, Resources, Project administration, Funding acquisition, Conceptualization.

## Data availability statement

The datasets used and analysed in this study are available from the corresponding author on reasonable request.

## Ethical statement

The studies included were approved by the ethical committee of Comité d’Ethique de la Recherche de l’Institut des Sciences Biomédicales Appliquées (CER-ISBA) under reference numbers 81/CER-ISBA (04/02/2016) and 197/CER-ISBA (05/04/2024). Additionally, the present molecular analysis was approved by the Ethics Committee of the Faculty of Medicine, University of Tübingen, Germany (Project No. 704/2022BO2). Written informed consent was obtained from the parents or guardians of all participating children, and assent was obtained from children aged 8 years or older. In addition, the consent form signed by each participant included permission for future malaria-related analysis using the collected samples.

## Declaration of generative AI and AI-assisted technologies in the manuscript preparation process

The Grammarly application was used to help improve the flow and structure. After using this tool, the author reviewed and edited the content as needed and takes full responsibility for the published article. However, we did not use generative AI (GenAI) tools to generate text, data, or graphics, or to assist with study design, data collection, analysis, or interpretation.

## Funding

This project is co-funded by the EDCTP2 Programme (RIA2018SD-2306), which is supported by the European Union, and the Swedish International Development Cooperation Agency (Sida). The views and opinions of authors expressed herein do not necessarily state or reflect those of EDCTP. We acknowledge support by the Open Access Publishing Fund of the University of Tübingen.

## Declaration of competing interest

The authors declare the following financial interests/personal relationships which may be considered as potential competing interests: Jana Held reports financial support was provided by the Open Access Publishing Fund of the University of Tübingen. Francis Emmanuel Towanou Bohissou reports financial support was provided by European and Developing Countries Clinical Trials Partnership. If there are other authors, they declare that they have no known competing financial interests or personal relationships that could have appeared to influence the work reported in this paper.
