# Supplementary material for: Changes in molecular markers of Plasmodium falciparum drug resistance between 2017 and 2024 in Southern Benin
Source: Int J Parasitol Drugs Drug Resist. 2026 Sep 1;32:100670. doi: 10.1016/j.ijpddr.2026.100670 (PMC13571362; doi:10.1016/j.ijpddr.2026.100670)
Supplement: Supplementary file 1 [file mmc1.docx]

Supplementary documents

**Table 1: WHO classification of *PfKelch13* markers associated with artemisinin partial resistance** (<https://www.who.int/tools/compendium-of-molecular-markers-for-antimalarial-drug-resistance>)

| **Validated marker (Epi., Lab. & Clin.)** | F446I |
| --- | --- |
|  | N458Y |
|  | C469Y |
|  | M476I |
|  | Y493H |
|  | G533S |
|  | R539T |
|  | I543T |
|  | P553L |
|  | R561H |
|  | P574L |
|  | C580Y |
|  | R622I |
|  | A675V |
| **Candidate marker (Epi. & Clin.)** | E252Q |
|  | P441L |
|  | C469F |
|  | N537I |
|  | G538V |
|  | V568G |
| **Potential marker (Lab.)** | P413A |
|  | F495L |
|  | R515K |
|  | M579I |
|  | D584V |
| **Potential marker (Clin.)** | P527H |
|  | G625R |
| **Potential marker (Epi.)** | K438N |
|  | G449A |
|  | T508N |
|  | Y511H |
|  | S577L |
|  | A582V |
|  | N585K |
|  | Q613E |

**Lab.**: Laboratory; **Clin.**: Clinical; **Epi.**: Genetic epidemiology

**Table 2**: **Single-nucleotide polymorphisms in *Pfcrt*, *Pfmdr1*, *Pfdhfr* and *Pfdhps* genes associated with antimalarial drug resistance.**

| **Gene** | **Nucleotide locus (Codon AA)** | **Wild Type** | | **Mutant** | |
| --- | --- | --- | --- | --- | --- |
|  |  | **Nucleotide** | **AA** | **Nucleotide** | **AA** |
| *Pfcrt* | 215 (72) | g | C | **c** | **S** |
|  | 219 (73) | a | V | **c** | V |
|  | 222 (74) | g | M | **t** | **I** |
|  | 223 (75) | a | N | **g** | **E** |
|  | 227 (76) | a | K | **c** | **T** |
| *Pfmdr1* | 256 (86) | a | N | **t** | **Y** |
|  | 257 (86) | a | N | **t** | **Y** |
|  | 551 (184) | a | Y | **t** | **F** |
|  | 3100 (1034) | a | S | **t** | **C** |
|  | 3101 (1034) | g | S | **c** | **C** |
|  | 3124 (1042) | a | N | **g** | **D** |
|  | 3736 (1246) | g | D | **t** | **Y** |
| *Pfdhfr* | 148 (50) | t | C | **c** | **R** |
|  | 152 (51) | a | N | **t** | **I** |
|  | 175 (59) | t | C | **c** | **R** |
|  | 323 (108) | g | S | **a** | N |
|  | 490 (164) | a | I | **t** | **L** |
| *Pfdhps* | 1291 (431) | a | I | **g** | **V** |
|  | 1306 (436) | t | S | **g** | **A** |
|  | 1307 (436) | c | S | **t** | **F** |
|  | 1310 (437) | c | A | **g** | **G** |
|  | 1618 (540) | a | K | **g** | **E** |
|  | 1742 (581) | c | A | **g** | **G** |
|  | 1837 (613) | g | A | **t** | **S** |

AA: amino acid, mutated nucleotide in bold.

Table 3: Sequencing success rate of molecular markers associated with antimalarial resistance in P. falciparum samples collected in 2017 and 2024 in southern Benin.

| **Variable** | **N** | **Total** | **2017**, N = 69 | **2024**, N = 289 |
| --- | --- | --- | --- | --- |
| ***PfKelch13*** | 358 | 281 (78.5%) | 25 (36.2%) | 256 (88.6%) |
| ***Pfdhfr*** | 358 | 344 (96.1%) | 66 (95.7%) | 278 (96.2%) |
| **Part 1_*Pfdhps*** | 358 | 335 (93.6%) | 60 (87.0%) | 275 (95.1%) |
| **Part 2_*Pfdhps*** | 358 | 300 (83.8%) | 60 (87.0%) | 240 (83.1%) |
| ***Pfdhps (both Part 1 and Part 2)*** | 358 | 291 (81.3%) | 60 (87.0%) | 231 (79.9%) |
| **Part 1_ *Pfmdr1*** | 358 | 354 (98.9%) | 69 (100%) | 285 (98.6%) |
| **Part 2_*Pfmdr1*** | 358 | 340 (95.0%) | 65 (94.2%) | 275 (95.1%) |
| ***Pfmdr1 (both Part 1 and Part 2)*** | 358 | 338 (94.4%) | 65 (94.2%) | 273 (94.5%) |

**Table 4: Single-nucleotide polymorphisms identified in the sample sequenced for the *PfKelch13* gene in Benin**

| **Gene** | **Nucleotide Locus (Codon AA)** | **Wild Type** | | **Mutant** | |
| --- | --- | --- | --- | --- | --- |
|  |  | **Nucleotide** | **AA** | **Nucleotide** | **AA** |
| *PfKelch13* | 1320 (440) | a | L | **g** | L |
|  | 1407 (469) | c | C | **t** | C |
|  | 1471 (491) | t | F | **c** | **L** |
|  | 1473 (491) | c | F | **t** | F |
|  | 1618 (540) | a | I | **g** | **V** |
|  | 1644 (548) | c | G | **t** | G |
|  | 1732 (578) | g | A | **t** | **S** |
|  | 1767 (589) | c | V | **a** | V |
|  | 1805 (602) | a | E | **g** | G |
|  | 1839 (613) | a | Q | **t** | **H** |
|  | 1861 (621) | g | A | **t** | **S** |
|  | 2026 (676) | g | A | **t** | **S** |

AA: amino acid, mutated nucleotide in bold.

**Table 5: Synonymous single-nucleotide polymorphisms identified in the sample sequenced for the *PfKelch13* gene in 2017 and 2024 in Benin**

| ***PfKelch13*** | **Mutation** | **2017** | **2024** |
| --- | --- | --- | --- |
| **Synonymous** | F491F | 1 (4.0%) | 0 (0%) |
|  | V589V | 0 (0%) | 1 (0.4%) |
|  | C469C | 0 (0%) | 3 (1.2%) |
|  | L440L, C469C, | 0 (0%) | 1 (0.4%) |
|  | G548G | 0 (0%) | 1 (0.4%) |

**Table 6: Prevalence of mutations, mixed infection and wild type at *Pfcrt and Pfmdr1* from samples collected in 2017 and 2024 in southern Benin.**

| **Variable** | **N** | **Total** | **2017**, N = 69 | **2024**, N = 289 |
| --- | --- | --- | --- | --- |
| ***Pfcrt* haplotype** | 352 |  |  |  |
| CVIET |  | 83 (23.6%) | 48 (71.7%) | 35 (12.3%) |
| CVMNK |  | 192 (54.5%) | 8 (11.9%) | 184 (64.6%) |
| CVMNK/CVIET |  | 77 (21.9%) | 11 (16.4%) | 66 (23.1%) |
| ***Pfmdr1* N86Y** | 354 |  |  |  |
| Mixed |  | 8 (2.3%) | 1 (1.5%) | 7 (2.5%) |
| Mutant |  | 17 (4.8%) | 7 (10.1%) | 10 (3.5%) |
| Wild Type |  | 329 (92.9%) | 61 (88.4%) | 268 (94.0%) |
| ***Pfmdr1* Y184F** | 354 |  |  |  |
| Mixed |  | 67 (18.9%) | 14 (20.3%) | 53 (18.6%) |
| Mutant |  | 164 (46.3%) | 31 (44.9%) | 133 (46.7%) |
| Wild Type |  | 123 (34.8%) | 24 (34.8%) | 99 (34.7%) |
| ***Pfmdr1* S1034C** | 340 |  |  |  |
| Wild Type |  | 340 (100%) | 65 (100%) | 275 (100%) |
| ***Pfmdr1* N1042D** | 340 |  |  |  |
| Wild Type |  | 340 (100%) | 65 (100%) | 275 (100%) |
| ***Pfmdr1* D1246Y** | 340 |  |  |  |
| Mixed |  | 1 (0.3%) | 0 (0%) | 1 (0.4%) |
| Wild Type |  | 339 (99.7%) | 65 (100%) | 274 (99.6%) |
| ***Pfmdr1* haplotype** | 338 |  |  |  |
| NFD |  | 206 (61.0%) | 37 (56.9%) | 169 (61.9%) |
| NF**Y** |  | 1 (0.3%) | 0 (0%) | 1 (0.4%) |
| N**Y**D |  | 107 (31.6%) | 20 (30.8%) | 87 (31.9%) |
| **YF**D |  | 21 (6.2%) | 7 (10.8%) | 14 (5.1%) |
| **Y**YD |  | 3 (0.9%) | 1 (1.5%) | 2 (0.7%) |

**Table 7:** **Prevalence of mutations, mixed infections and wild type at** ***Pfdhfr and Pfdhps* from samples collected in 2017 and 2024 in southern Benin.**

| **Variable** | **N** | **Total** | **2017**, N = 69 | **2024**, N = 289 |
| --- | --- | --- | --- | --- |
| ***Pfdhfr* C50R** | 344 |  |  |  |
| Wild Type |  | 344 (100%) | 66 (100%) | 278 (100%) |
| ***Pfdhfr* N51I** | 344 |  |  |  |
| Mix |  | 5 (1.5%) | 1 (1.5%) | 4 (1.4%) |
| Mutant |  | 328 (95.3%) | 63 (95.5%) | 265 (95.3%) |
| Wild Type |  | 11 (3.2%) | 2 (3.0%) | 9 (3.2%) |
| ***Pfdhfr* C59R** | 344 |  |  |  |
| Mix |  | 10 (2.9%) | 1 (1.5%) | 9 (3.2%) |
| Mutant |  | 333 (96.8%) | 65 (98.5%) | 268 (96.4%) |
| Wild Type |  | 1 (0.3%) | 0 (0%) | 1 (0.4%) |
| ***Pfdhfr* S108N** | 344 |  |  |  |
| Mix |  | 2 (0.6%) | 1 (1.5%) | 1 (0.4%) |
| Mutant |  | 342 (99.4%) | 65 (98.5%) | 277 (99.6%) |
| ***Pfdhfr* I164L** | 344 |  |  |  |
| Wild Type |  | 344 (100%) | 66 (100%) | 278 (100%) |
| ***Pfdhps* I431V** | 335 |  |  |  |
| Mix |  | 24 (7.2%) | 0 (0%) | 24 (8.7%) |
| Mutant |  | 10 (3.0%) | 5 (8.3%) | 5 (1.8%) |
| Wild Type |  | 301 (89.8%) | 55 (91.7%) | 246 (89.5%) |
| ***Pfdhps* S436A/F** | 335 |  |  |  |
| Mix |  | 55 (16.4%) | 2 (3.3%) | 53 (19.3%) |
| Mutant |  | 57 (17.0%) | 15 (25.0%) | 42 (15.3%) |
| Wild Type |  | 223 (66.6%) | 43 (71.7%) | 180 (65.4%) |
| ***Pfdhps* A437G** | 335 |  |  |  |
| Mix |  | 8 (2.4%) | 1 (1.7%) | 7 (2.5%) |
| Mutant |  | 321 (95.8%) | 58 (96.6%) | 263 (95.7%) |
| Wild Type |  | 6 (1.8%) | 1 (1.7%) | 5 (1.8%) |
| ***Pfdhps* K540E** | 300 |  |  |  |
| Wild Type |  | 300 (100%) | 60 (100%) | 240 (100%) |
| ***Pfdhps* A581G** | 300 |  |  |  |
| Mix |  | 11 (3.7%) | 0 (0%) | 11 (4.6%) |
| Mutant |  | 13 (4.3%) | 5 (8.3%) | 8 (3.3%) |
| Wild Type |  | 276 (92.0%) | 55 (91.7%) | 221 (92.1%) |
| ***Pfdhps* A613S** | 300 |  |  |  |
| Mix |  | 3 (1.0%) | 0 (0%) | 3 (1.3%) |
| Mutant |  | 25 (8.3%) | 7 (11.7%) | 18 (7.5%) |
| Wild Type |  | 272 (90.7%) | 53 (88.3%) | 219 (91.2%) |

**Table 8: Geometric mean parasitemia analysed by molecular marker group from samples collected in southern Benin in 2017 and 2024.**

| Marker | Variant | n | GMPD | Median_IQR | Range | Test | p_value (Bonferroni Correction) |
| --- | --- | --- | --- | --- | --- | --- | --- |
| ***PfKelch13*** | Mutant | 17 | 359.3 | 305 (48–904) | 39.5–417906.5 | Wilcoxon rank-sum test | 0.939 |
|  | Wild Type | 264 | 315.2 | 186.2 (56–1049.2) | 16–69881 |  |  |
| ***Pfcrt*** | CV**IET** | 83 | 516.2 | 325 (70–2127.5) | 32–69881 | Wilcoxon rank-sum test |  |
|  |  |  |  |  |  |  | **0.0047 (0.019)** |
|  |  |  |  |  |  |  |  |
|  | CVMNK | 192 | 241.8 | 109.8 (48–810.9) | 16–417906.5 |  |  |
| ***Pfmdr1*** | N**F**D | 206 | 344.3 | 248.2 (57.8–1113.8) | 16–417906.5 | Wilcoxon rank-sum test | 0.175 |
|  | NYD | 107 | 254.1 | 110 (56–831.2) | 23.5–69881 |  |  |
| ***Pfdhfr/ Pfdhps*** | **IRN**I/I**AG**KAA | 58 | 340.1 | 209.2 (64–1125.2) | 23–20712 | Kruskal-Wallis test | 0.125 |
|  | **IRN**I/IS**G**KAA | 157 | 471.0 | 305 (64–2238.5) | 16–417906.5 |  |  |
|  | **IRN**I/**VAG**KAA | 10 | 115.3 | 48 (42–297.9) | 23–4807 |  |  |
|  | **IRN**I/**VAG**K**G**S | 10 | 648.0 | 925 (353–1592.5) | 30–11889 |  |  |

GMPD: Geometric Mean Parasite Density; IQR: Interquartile Range

**Table 9: Fever prevalence by molecular marker group from samples collected in southern Benin in 2017 and 2024.**

| Marker | Variant | n | Fever | Test | p_value |
| --- | --- | --- | --- | --- | --- |
| ***PfKelch13*** | Mutant | 17 | 2/17 (11.8%) | Fisher test | 0.681 |
|  | Wild Type | 264 | 26/264 (9.8%) |  |  |
| ***Pfcrt*** | CV**IET** | 83 | 11/83 (13.3%) | Fisher test | 0.393 |
|  | CVMNK | 192 | 18/192 (9.4%) |  |  |
| ***Pfmdr1*** | N**F**D | 206 | 24/206 (11.7%) | Fisher test | 0.326 |
|  | NYD | 107 | 8/107 (7.5%) |  |  |
| ***Pfdhfr/Pfdhps*** | **IRN**I/I**AG**KAA | 58 | 6/58 (10.3%) | Fisher test | 0.915 |
|  | **IRN**I/IS**G**KAA | 157 | 18/157 (11.5%) |  |  |
|  | **IRN**I/**VAG**KAA | 10 | 1/10 (10%) |  |  |
|  | **IRN**I/**VAG**K**GS** | 10 | 0/10 (0%) |  |  |
